# Joint Cultivation of *Allium ursinum* and *Armoracia rusticana* under Foliar Sodium Selenate Supply

**DOI:** 10.3390/plants11202778

**Published:** 2022-10-20

**Authors:** Zarema Amagova, Visita Matsadze, Zulfia Kavarnakaeva, Nadezhda Golubkina, Marina Antoshkina, Agnieszka Sękara, Alessio Tallarita, Gianluca Caruso

**Affiliations:** 1 Chechen Scientific Institute of Agriculture, Lilovaya 1, 366021 Grozny, Russia; 2 Federal Scientific Center of Vegetable Production, Selectsionnaya 14, VNIISSOK, Odintsovo District, 143072 Moscow, Russia; 3 Department of Horticulture, Faculty of Biotechnology and Horticulture, University of Agriculture, 31-120 Krakow, Poland; 4 Department of Agricultural Sciences, University of Naples Federico II, Naples, 80055 Portici, Italy

**Keywords:** allelopathy, horseradish, ramson, selenium

## Abstract

Despite the high value of ramson (*Allium ursinum*) in medicine and nutrition, it is not cultivated in open fields due to the need for shading as well as weeding during the early crop stages. Research was carried out in an open field with the aim to improve *A. ursinum* growth, through its intercropping with *Armoracia rusticana* (horseradish). In the latter context, with and without sodium selenate application, ramson and horseradish showed reciprocal growth stimulation, as ramson biomass increased by 1.28 times and horseradish root biomass by 1.7 times. The biofortification level of horseradish roots increased from 5.9 to 9.6 times due to joint plant growth under selenium (Se) supply. The opposite phenomenon was recorded for ramson leaves, as the biofortification level decreased from 11.7 in the case of Se supplementation to 6.7 in plants supplied with sodium selenate when jointly cultivated with horseradish. Among the tested antioxidants, the highest increase due to joint cultivation and/or Se supply was recorded for ascorbic acid by 1.69 times in ramson leaves and 1.48 and 1.37 times in horseradish roots and leaves, respectively. All treatments significantly increased the total antioxidant activity (AOA) of horseradish leaves (by 1.33–1.49 times) but not roots. Comparison of the results obtained in field conditions with those obtained earlier for the Se biofortification of ramson in the natural habitat (forest) revealed significantly higher levels of the plant’s antioxidant status under environmental stress (field) and a decrease in the correspondent differences as a consequence of Se biofortification. The estimation of allelopathic beneficial interaction between ramson and horseradish implies the efficiency of ramson growth and production of functional food with high levels of Se (Se–ramson leaves and Se–horseradish roots).

## 1. Introduction

Among edible herbs, ramson (*Allium ursinum*) occupies a special place due to its powerful biological activity and unique taste [1,2]. This plant is widely used in medicine for insulin level and blood pressure normalization, as an anti-sclerotic, anti-microbial, and anti-inflammatory agent, efficient in treatment of cold, fever, bronchitis, and ulcer, and used also for wound healing [3,4,5,6].

In Romania, a food additive based on biologically active ramson is commercially produced for organism detoxification, cholesterol level normalization, and memory strengthening [7]. Intensive ramson growth in early spring determines a possibility of quick replenishment of vitamin deficiency in the human body, including vitamin C and other antioxidants, and improves immunity [8]. This plant was shown to be tolerant to high Se concentrations and may be attributed to a group of plants, indicators of the element [9]. Attempts to obtain ramson biofortified with Se revealed the high responsiveness of the plant to foliar selenate supply leading to the enhancement of the total antioxidant status and nutritional value of the product [9]. This product is highly demanded as a valuable source of biologically active Se and a natural antioxidant in prophylactics and organism protection against cardiac, oncological, and viral diseases, including COVID-19 [10], and to normalize brain activity and reproductive function [11].

Ramson does not tolerate bright light and drought and grows most successfully in deciduous forests on wet, lightly acidic soils with high organic matter content [12]. The distribution range of this plant includes Northern America, Africa, Europe, Minor Asia, Caucasus, and Siberia up to Kamchatka [13]. Natural resource depletion and the high demand for ramson for medicinal and food purposes entail the necessity of the development of efficient plant growth technology as an agricultural crop and its biofortification with Se.

In this respect, the search for plants capable to provide sufficient shade for ramson and improve its growth due to the beneficial allelopathic effect is highly attractive. The tolerance of horseradish to environmental stresses, weed growth inhibition due to glucosinolate and their hydrolysis product, especially sinigrin, release to soil [14,15], and the sufficient shade of a relatively large area make the joint cultivation of ramson and horseradish highly interesting with or without foliar Se supply. Despite the risk of this approach, a successful result could provide an opportunity for both ramson growth in the field and functional food production with high levels of Se, both of Se-enriched ramson and horseradish. Indeed, *Allium* and Brassicaceae vegetables are known to synthetize methylated forms of Se-containing amino acids and peptides, possessing high anti-carcinogenic activity [11,16]. Furthermore, cruciferous representatives including horseradish also synthetize Se-containing glucosinolates with anti-cancer activity significantly higher than ordinary glucosinolates lacking Se [17,18]. On the other hand, literature reports indicate strong allelopathic properties, even ramson’s ability to inhibit the growth of forest grasses and legumes [13] via soil emission of polyphenols, such as p-coumaric, ferulic, p-hydroxybenzoic, vanillic, and syringic acids, inhibiting the growth of the neighboring species.

The aim of the present study was the evaluation of the field growth efficiency of ramson intercropped with horseradish as a plant providing shade with or without Se supply.

## 2. Results and Discussion

### 2.1. Morphological Characteristics

**Figure 1:**
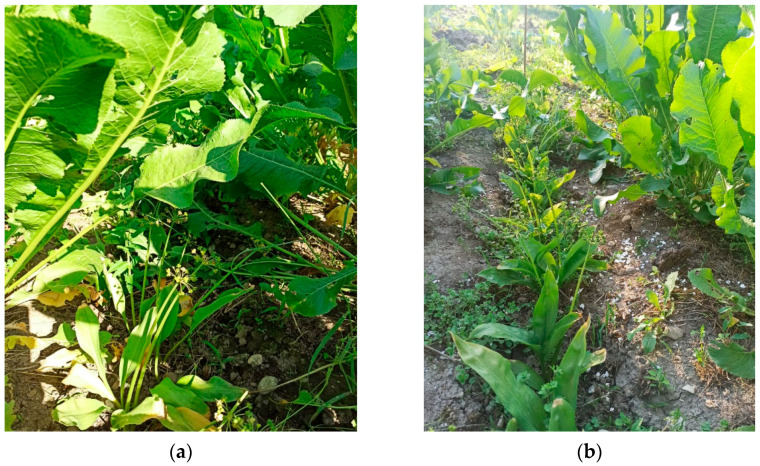
Experimental beds of joint ramson–horseradish cultivation: (**a**) shading effect of horseradish and (**b**) ramson appearance on experimental beds.

Intercropping of ramson and horseradish provided favorable conditions for both species (Figure 1). Indeed, the tolerance of horseradish to drought and moderate resistance to pest attack [19] represent important properties of this plant, improving ramson protection against weeds, herbivory, and intensive sun radiation.

The lack of growth inhibition of horseradish intercropped with ramson makes it adopt the tolerance of the former to water-soluble polyphenol compounds of ramson leaves freely released by the leaves falling at the beginning of summer [13]. Several phenolic compounds and volatile sulfur derivatives of ramson bulbs, known to suppress surrounding plant growth [2,13], do not reveal harmful effects on horseradish development.

Accordingly, sinigrin of horseradish roots and leaves, known to inhibit the neighboring plant growth and advised for weed control [20], is not harmful to ramson.

As can be seen in Table 1, the joint cultivation of ramson and horseradish did not affect significantly either ramson leaf number and area, plant height, or leaf biomass and yield, though there was a tendency for the parameters to increase. On the contrary, a more pronounced beneficial effect was recorded for horseradish demonstrating a 27% increase in the aboveground biomass and a 65.7% increase in plant root biomass as a result of joint cultivation.

In these conditions, plant biofortification with Se is especially attractive due to the active substitution of sulfur in *Allium* and *Brassica* species with its analog Se [21]. Indeed, the high tolerance of Allium and Brassica plants to Se and the ability to synthetize methylated forms of Se-containing amino acids and peptides (SeMeSeCys, γ-glutamyl SeMeSeCys), known to show powerful anti-carcinogenic effects [16,22], provide a good opportunity for successful biofortification of plants with this essential element to humans.

The beneficial effect of low Se doses on plant growth and development was previously described for many plant species including representatives of the Brassicaceae and Amaryllidaceae families [9,18,23]. In the case of ramson and horseradish joint cultivation, Se supplementation improved the growth of both plants. Indeed, compared to control plants, ramson height increased by 16.9%, leaf area by 32.4%, plant biomass by 27.7%, and yield by 16.8%. A similar phenomenon, but with a more valuable beneficial effect, occurred with horseradish. Indeed, the data in Table 1 indicate that joint ramson–horseradish cultivation under Se supply resulted in an increase in horseradish height by 6.2%, leaf width by 20%, plant total biomass by 70.7%, plant root biomass by 71.4%, and aboveground plant biomass by 70.6%. The evaluation of the growth beneficial effect between different treatments of *A. rusticana* (Table 1) suggested that Se supplementation did not affect leaf biomass in horseradish plants, while biofortification of jointly cultivated plants resulted in a similar 70% increase in both the leaf and root biomass of horseradish (Table 2).

It is known that *A. ursinum* influences other herbaceous plants in the plant community via soil and volatile compounds which inhibit seed germination and plant growth [13]. The present results are the first example of the *A. ursinum* beneficial effect.

As far as horseradish is concerned, the data regarding the allelopathic properties of this plant are more controversial. High levels of glucosinolates in horseradish and their hydrolysis products are known to be crucial in allelopathy [24]. Indeed, separate data indicate its beneficial effect: 5% and 15% dilution of aqueous extracts from horseradish metamorphosed roots stimulated kernel germination ability in cereal species and accumulation of the fresh and dry biomass in triticale and wheat, but not in the barley [25]. The authors reported a dose-dependent phenomenon and strong allelopathic properties of root isothiocyanates. Other investigations demonstrated a strong inhibition effect of glucosinolate sinigrin—allyl isothiocyanates, a horseradish root hydrolysis product—on weed and lettuce seed growth [19]. Furthermore, the investigation of Simpson et al. [26] demonstrated a strong growth inhibition effect of horseradish extract in onion. As all the above-mentioned works deal with horseradish extracts, it may be supposed that the inhibition could relate to high doses of glucosinolates. The present results provide less glucosinolate leaching from roots, thus eliciting growth, though more intensive investigations are necessary to prove this hypothesis.

### 2.2. Biochemical Characteristics

Analysis of nitrates and dry matter content in ramson and horseradish tissues indicated a lack of significant effect on the parameters, either of intercropped plants or plants supplemented with Se (Table 3). The only exception is represented by the high nitrate level in horseradish roots under joint plant cultivation. Nevertheless, a 22% increase in nitrate concentration in horseradish roots under joint cultivation may be partly attributed to high nitrate variations during plant growth and development.

The accumulation of antioxidants by ramson and horseradish is especially important, as reported in Table 3 for total antioxidant activity (AOA) and contents of phenolics (TP) and ascorbic acid (AA), the latter showing the highest increase of 1.69 times in ramson leaves and 1.48 times and 1.37 times in horseradish roots and leaves, respectively. It is important that all treatments (Se biofortification, joint cultivation, and Se supply under joint cultivation) provided similar effects on AA increase in ramson leaves and horseradish roots and leaves (Table 3 and Table 4). Participation of AA in phytohormone biosynthesis, control of the division, elongation, and differentiation of cells, and antioxidant defense [27,28] indicates the significance of the recorded phenomenon of AA increase in ramson and horseradish growth and development.

As far as fat-soluble antioxidants are concerned, their content expressed as AOA is mostly affected in horseradish leaves both by the joint cultivation of plants and Se supply (Table 4). Indeed, while this parameter increased by 1.33–1.49 times in horseradish leaves, only a 1.11–1.16 times increase was recorded for ramson leaf AOA under joint cultivation and Se supply (Table 3).

Participation of Se in carbohydrate metabolism is known to stimulate plant antioxidant defense and improve the nutritional quality of the product [29]. In this respect, a significant increase in monosaccharide content in horseradish roots as a result of Se supply represents an important characteristic of horseradish, suggesting the existence of a disaccharide hydrolysis process (Table 4). Indeed, taking into account the stability of total sugar content in horseradish roots, a decrease in the di: monosaccharides ratio according to the following sequence may be indicated: control (3.29) > joint cultivation (2.91) > joint cultivation under Se supply (1.81) > Se (1.28).

**Figure 2:**
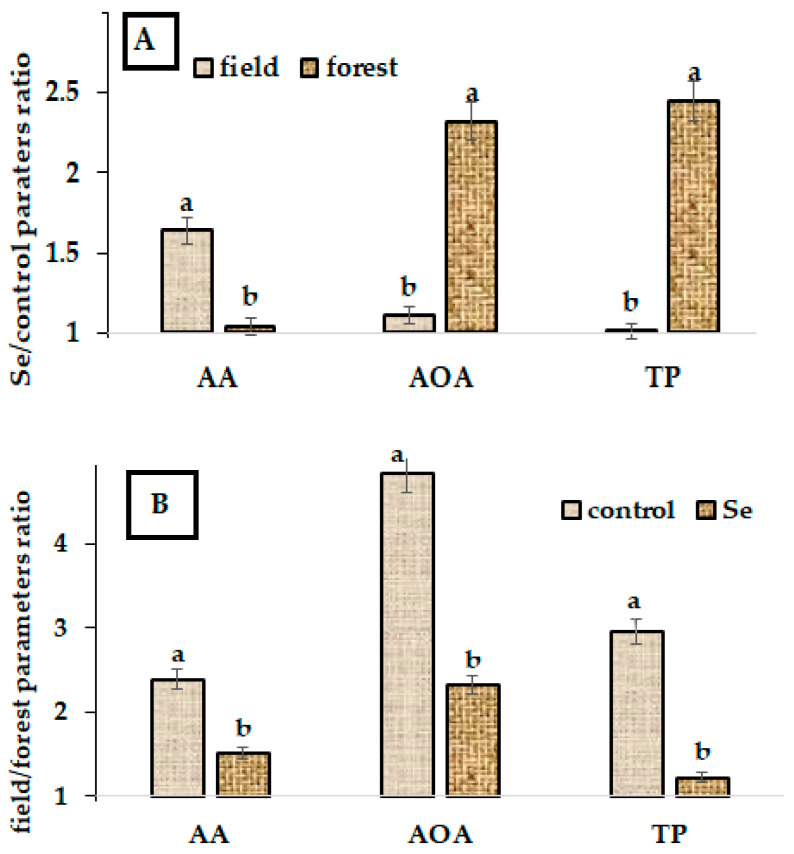
Ascorbic acid (AA), polyphenol content (TP), and total antioxidant activity (AOA) changes in ramson leaves as affected by (**A**) Se supply (Se fortified/control) and (**B**) place of habitat (field/forest). For each parameter, values with different letters differ statistically according to Duncan test at *p* < 0.05.

The comparison of the obtained results with the data of an earlier attempt at the foliar Se supply of ramson grown in the natural habitat (forest) [9] revealed that in field conditions, Se supply resulted in a significant AA accumulation, while a negligible effect was recorded in the forest. On the contrary, Se biofortification provided a higher beneficial effect for AOA and TP levels in plants grown in the forest (Figure 2A,B).

Furthermore, control plants in field conditions demonstrated significantly higher antioxidant status (AA, AOA, and TP) compared to plants grown in the forest, which indicates indirectly the existence of increased oxidant stress for ramson in the field. Data presented in Figure 2B indicate that Se supplementation decreased the differences in the antioxidant indicators of plants grown in forest and field conditions.

### 2.3. Se Accumulation

Taking into account that both *Allium* and cruciferous vegetables are regarded as Se accumulators tolerant to Se supplementation [18,23,30], it should be highlighted that, while Se biofortification of wild ramson has been investigated previously [9], so far, no attempts have been made on the production of *A. rusticana* roots fortified with Se, though this approach for human Se status optimization is highly valuable as horseradish roots are a well-known spice with significant antioxidant activity and high medicinal value [31].

**Figure 3:**
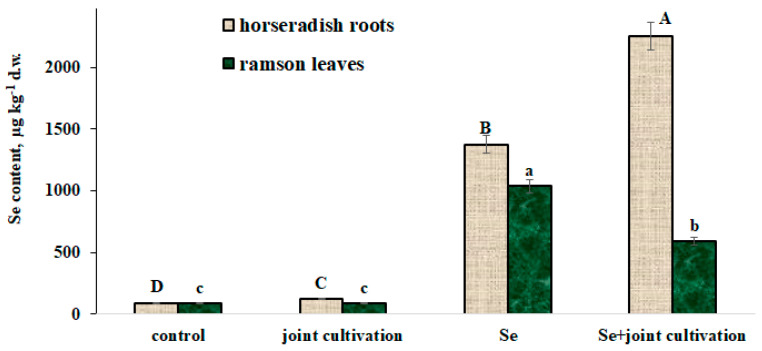
Effect of separate and joint cultivation of ramson and horseradish under Se supply on plant Se accumulation. Values with similar letters do not differ statistically according to Duncan test at *p* < 0.05.

Se biofortification of plants grown separately or intercropped revealed unusual peculiarities (Figure 3): intercropping of plants in ordinary conditions without Se supply resulted in a significant Se content increase in horseradish roots, demonstrating no effect on ramson leaf Se levels.

A more pronounced effect was recorded for plants fortified with this microelement. Indeed, the data shown in Figure 2 indicate an increase in the Se biofortification level of horseradish roots from 5.88 (separate Se application) to 9.63 (under joint cultivation) and the opposite phenomenon of the ramson leaf Se biofortification level (the latter decreased from 11.78 to 6.70). Furthermore, higher concentrations of Se in horseradish may be attributed to the higher leaf area stimulating the accumulation of the element. On the other hand, the phenomenon may reflect a new example of allelopathic interactions between ramson and horseradish, similar to those described for Se hyperaccumulators (*Stanleya pinnata* and *Astragalus bisulcatus*), known to increase the neighboring plants’ Se status [32]. We recorded elemental allelopathy for other elements previously in *Artemisia scoparia* and radish grown under Pb supply [33].

From a practical point of view, both Se-fortified plants may be considered as new functional food products. Indeed, the suggested traditional use for cold and respiratory infections of horseradish roots is about 20 g per day, which corresponds to 9.3 µg Se. Taking into account the recommended dietary allowance (RDA) of Se consumption for adult males and females (55–75 µg day^−1^) [34], the calculated value will correspond to 12.4–16.9 % of RDA value. As far as ramson is concerned, consumption of 50 g of leaves will correspond to 4.4–6.0% of Se RDA. The latter values are not high, but in general, the proposed technology of ramson–horseradish plant intercropping provides the first possibility of industrial ramson growth.

It should be also highlighted that the possibility to increase antioxidant and anti-carcinogenic properties of ramson and horseradish due to Se supply [35,36,37] is able to synthetize significant amounts of Se-containing methylated amino acids and Se-containing glucosinolates with powerful anti-carcinogenic properties and a lack of glucosinolate biosynthesis suppression under moderate Se concentration supply. On the other hand, special investigations are necessary to reveal the effect of the proposed conditions on glucosinolate accumulation in horseradish.

## 3. Materials and Methods

### 3.1. Object of Investigation and Cultivation Conditions

The experiment started in November, both in 2020 and 2021. The experimental plot was 6 m × 4 m with three replicates. The distance between rows of horseradish–ramson was 50 cm, and between plants along each row, it was 50 cm for horseradish and 20 cm for ramson. Plants were grown on different levels: *A. ursinum* was planted in a trench 20 cm × 20 cm × 4000 cm, 0.2 m below the horseradish level. The bottom of a trench was covered with 10 cm humus composition of the topsoil. *A. ursinum* bulbs were harvested in a forest of Nozhay-Yurtovsky region of the republic (Sim-Sir settlement, 43°00′34′′ N; 46°27′57′′ E), placed on the humus layer with 20 cm between each bulb on 13.11.2020, covered by 10 cm of soil, and watered with 10 L per each trench. After watering, each trench was covered with 10 cm of autumn leaves. No fertilizers, insecticides, herbicides, or fungicides were used in the experiment. In spring, experimental beds were shaded to avoid sunburn of horseradish and *A. ursinum* leaves as well as to bring the conditions of the experiment closer to natural ones. An 80% shading net, with cells of 1.5 cm × 1.5 cm, was used as a protective coating. This level of shading turned out to be optimal for both plants, which was especially evident during the period of high solar intensity in July–August.

Horseradish was planted in cuttings, 20–25 cm tall and with 1–1.5 cm diameter, with the lower part cut obliquely.

Chernozem was first poured into each hole, and the cuttings were watered with water at room temperature, deepened into the soil, strongly trampled, and mulched with 10 cm black soil for 2 cm foliage. The rows of horseradish–ramson–horseradish–ramson were placed alternately in each plot.

The experiment with multi-level planting of these plants aimed both to establish their symbiosis and to use a large area of horseradish leaf surface as a shading plant for ramson.

Moreover, with different methods of propagation of ramson, it was noticed that before the enlargement and strengthening of ramson seedlings, weeds can represent a serious threat, until their complete suppression. Multi-level landing, as experience has shown, solved this problem by 80%.

Soil characteristics (Gera inc. production): a mixture of peat, sand, and complex mineral fertilizer, limestone flour (dolomite), containing (N)—250 mg kg^−1^ d.w.; (P_2_O_5_)—275 mg kg^−1^ d.w.; (K_2_O)—275 mg kg^−1^ d.w. with pH 5.5.

Foliar Se biofortification was performed three times for both crops at 10-day intervals using 0.05 g L^−1^ solution of sodium selenate: 7.05, 18.05, and 28.05.

Plants were harvested in the first 10 days of September in 2021 and at the end of May in 2022. Horseradish roots were washed to remove soil. All samples were cut into small pieces, dried at room temperature to constant weight, and homogenized.

Mean temperature and rainfall during vegetation period are reported in Table 5.

### 3.2. Biochemical Analysis

#### 3.2.1. Dry Matter

The dry residue was assessed gravimetrically by drying the samples in an oven at 70 °C until constant weight. The calculation of dry matter content was performed according to the formula:

D.M. (%) = (M_1_:M_2_) × 100,

where M_1_ is the sample biomass after drying, and M_2_- is the sample biomass before drying.

#### 3.2.2. Ascorbic Acid

It was determined by visual titration of plant extracts in 6% trichloracetic acid with Tillman’s reagent [38]. Two grams of fresh ramson/horseradish leaves or horseradish root homogenates was ground in porcelain mortar with 5 mL of 6% trichloracetic acid and quantitatively transferred to a measuring cylinder. The volume was brought to 60 mL using trichloracetic acid, and the mixture was filtered through filter paper 15 min later. The concentration of ascorbic acid was determined from the amount of Tillman’s reagent that went into the titration of the sample up to pink color, which did not disappear within 20–30 s. The ascorbic acid (AA) content was calculated according to the formula:

AA (mg 100 g^−1^ d.w.) = {T × (V − 0.03) × 60 × 100}: (m × V_1_)

where T is the titer of Tillman’s reagent;

V is the volume of Tillman’s reagent needed for the titration of sample extract, in mL;

0.03 is the volume of reagent needed for the titration of blank sample (6% trichloroacetic acid solution), in mL;

60 is the total volume of the extract tested, mL;

100 is the conversion of the data per 100 g of a sample;

M is the sample biomass, in g;

V_1_ is the volume of the extract used for the determination.

The sensitivity of visual titration is equal to 5 µg mL^−1^.

#### 3.2.3. Preparation of Ethanolic Extracts

One gram of dry leaf/petiole/root powder was extracted with 20 mL of 70% ethanol (7:3, *v*/*v*) at 80 °C in 1 h. The mixture was cooled and quantitatively transferred to a volumetric flask, and the volume was adjusted to 25 mL. The mixture was filtered through filter paper and used further for the determination of polyphenols and total antioxidant activity.

#### 3.2.4. Polyphenols

Polyphenols were determined spectrophotometrically based on the Folin–Ciocalteu colorimetric method according to [39]. One mL of ethanolic extract prepared according to Section 3.2.3 was transferred to a 25 mL volumetric flask, to which 2.5 mL of saturated sodium carbonate solution and 0.25 mL of diluted (1:1, *v*/*v*) Folin–Ciocalteu reagent were added, and the volume was brought to 25 mL with distilled water. One hour later, the solutions were analyzed through a spectrophotometer (Unico 2804 UV, Dayton, NJ, USA), and the concentration of polyphenols was calculated according to the absorption of the reaction mixture at 730 nm. Gallic acid was used as an external standard, and the total polyphenols were expressed as mg of gallic acid equivalents per g (mg GAE g^−1^ d.w.) from the calibration curve (R^2^ = 0.997) using gallic acid. Gallic acid (0.5 g) was dissolved in 100 mL of distilled water in volumetric flask. The resulting solution was used to prepare 0, 50, 100, 250, 400, and 500 mg L^−1^ of gallic acid. The calibration curve was built using 0.5 mL of each probe after reaction with Folin–Ciocalteu reagent, similar to sample analysis.

#### 3.2.5. Antioxidant Activity (AOA)

The antioxidant activity of samples (roots, stems, and leaves) was assessed using a redox titration method according to Golubkina et al. [39] via titration of 1 mL 0.01 N potassium permanganate solution in 8 mL of distilled water and 1 mL of 20% sulphuric acid with ethanolic extracts, produced as described in Section 3.2.3. The reduction of potassium permanganate to colorless Mn^+2^ in this process reflects the quantity of antioxidants dissolvable in 70% ethanol. As an external standard, 0.02% gallic acid was used. The values were expressed in mg gallic acid equivalents (mg GAE g^−1^ d.w.).

#### 3.2.6. Nitrates

Nitrates were assessed using an ion-selective electrode with an ionomer Expert-001 (Econix, Russia). Five grams of fresh ramson/horseradish homogenates was mixed with 50 mL of distilled water. A quantity of 45 mL of the resulting extract was mixed with 5 mL of 0.5 M potassium sulfate background solution, necessary for regulating the ionic strength, and analyzed by the ionomer for nitrate determination. The results were expressed in g kg^−1^ DW.

#### 3.2.7. Mono- and Di-Saccharides

The monosaccharides were determined using the ferricyanide colorimetric method, based on the ability to reduce sugars when heated with an alkaline solution of potassium ferricyanide K_3_ Fe(CN)_6_ to reduce the latter into potassium ferrocyanide K_4_ Fe(CN)_6_ [40]. Two grams of dried homogenized sample was extracted with 150 mL of distilled water at 80 °C in half an hour. After cooling, 5 mL of lead acetate saturated solution was added to precipitate proteins, followed by 5 mL of 19% sodium sulfate solution to remove lead excess. The volume was adjusted to 250 mL with distilled water. The filtrate was used for titration of 10 mL of 1% potassium ferricyanide solution in the presence of 5 mL 1% sodium hydroxide and a drop of 1% methylene blue as an internal indicator until the entire fluid in the flask was decolorized. Each determination was performed in triplicate. The total sugars were analogically determined after acidic hydrolysis of water extracts with 20% hydrochloric acid. Fructose was used as an external standard. The results were expressed in % per d.w.

#### 3.2.8. Selenium

Selenium was analyzed using the fluorometric method previously described for tissues and biological fluids [41]. Dried homogenized samples were digested via heating with a mixture of nitric-perchloric acids, subsequent reduction of selenate (Se^+6^) to selenite (Se^+4^) with a solution of 6 N hydrochloric acid, and the formation of a complex between Se^+4^ and 2,3-diaminonaphtalene (Sigma-Aldrich, St. Louis, MO, USA). Calculation of the Se concentration (in µg kg^−1^ DW) was performed by recording the piazoselenol fluorescence value in hexane at 519 nm λ emission and 376 nm λ excitation with fluorimeter Fluorate 02–5M (Lumex, Saint Petersburg, Russia). Each determination was performed in triplicate. The precision of the results was verified using in each determination two reference standards: Se-fortified chervil stem powder and lyophilized cabbage powder, with Se concentration of 1865 μg kg^−1^ and 150 μg kg^−1^, respectively (Federal Scientific Vegetable Center, Moscow, Russia). The concentration of Se was determined using a calibration curve (R^2^ = 0.996) built with 5 different Se concentrations of sodium selenate (0; 0.1; 0.2; 0.4; 0.5; and 0.6 nM Se). The Se concentration is calculated according to the formula:

Se (kg^−1^ d.w.) = 79 × C: a,

where C is the Se content in a probe, determined from a calibration curve, nM;

79 is the Se atomic mass;

a is the sample mass, g.

### 3.3. Statistical Analysis

The data were processed by analysis of variance, and mean separations were performed through Duncan’s multiple range test, with reference to 0.05 probability level, using SPSS software version 21 (Armonk, NY, USA). Data expressed as percentages were subjected to angular transformation before processing.

## 4. Conclusions

Intercropping of ramson and horseradish under Se supply showed powerful beneficial allelopathic effects between the two plant species, which resulted in plant growth stimulation, antioxidant status improvement, and changes in Se biofortification levels. The revealed phenomenon suggests the possibility of industrial ramson cultivation via joint cultivation with horseradish and production of functional food products with high levels of Se. Further investigations are needed to clarify the chemical composition and nutritional value of ramson and horseradish biofortified with Se.

## Figures and Tables

**Table 1:** Morphological characteristics and yield of ramson (*Allium ursinum*).

Parameter	Control	Joint Cultivation	Se	Se + Joint Cultivation
Plant height (cm)	35.0 ± 1.3 b	39.1 ± 1.7 a	38.7 ± 1.5 a	40.9 ± 1.8 a
Leaf number	2.3 ± 0.3 a	2.6 ± 0.3 a	2.6 ± 0.3 a	2.7 ± 0.3 a
Leaf area (cm^2^)	89.3 ± 11.4 b	104.1 ± 15.5 ab	116.3 ± 10.2 a	118.2 ± 11.3 a
Plant biomass (g)	14.8± 1.1 b	17.4 ± 1.2 ab	16.8 ± 1.1 ab	18.9 ± 1.2 a
Yield (g m^−2^)	980 ±64.2 b	1069 ± 58.2 ab	1046 ± 68.1 ab	1145 ± 66.4 a

Along each line, values with the same letters do not differ statistically according to Duncan test at *p* < 0.05.

**Table 2:** Morphological characteristics and yield of horseradish (*Armoracia rusticana*).

Parameter	Control	Joint Cultivation	Se	Se + Joint Cultivation
Plant height, cm	89.0 ± 6.6 a	92.2 ± 7.4 a	88.7 ± 6.5 a	94.5 ± 7.2 a
Leaf width, cm	15.0 b	17.4 a	15.2 b	18.0 a
Plant total mass, g	290 ± 31 c	394 ± 32 b	320 ± 30 c	495 ± 38 a
Plant root mass, g	35 ± 2.5 c	58 ± 3.7 a	48 ± 4.1 b	60 ± 5.0 a
Plant leaf mass, g	255 ± 23 c	336 ± 31 b	272 ± 25 bc	435 ± 40 a
Aboveground plant mass, kg m^−2^	1.02 ± 0.08c	1.27 ± 0.10 b	1.09 ± 0.09 bc	1.74 ± 0.12 a

Along each line, values with the same letters do not differ statistically according to Duncan test at *p* < 0.05.

**Table 3:** Biochemical characteristics of ramson (*Allium ursinum*) leaves.

Parameter	Control	Joint Cultivation	Se	Se + Joint Cultivation
Dry matter, %	11.75 ± 1.1 a	11.0 ± 1.0 a	11.70 ± 1.1 a	11.1 ± 1.1 a
Nitrates, mg kg^−1^ d.w.	971 ± 78 a	983 ± 86 a	1023 ± 91 a	939 ± 90 a
Ascorbic acid, mg 100 g^−1^ d.w.	328 ± 2 b	555 ± 38 a	532 ± 43 a	543 ± 42 a
AOA *, mg GAE g^−1^ d.w.	41.1 ± 2.6 b	45.8 ± 2.8 ab	45.7 ± 2.8 ab	47.5 ± 3.1 a
TP **, mg GAE g^−1^ d.w.	15.7 ± 1.1 a	15.1 ± 1.0 a	15.9 ± 1.2 a	16.2 ± 1.2 a

* AOA: total antioxidant activity; ** TP: total polyphenol content. Along each line, values with the same letters do not differ statistically according to Duncan test at *p* < 0.05.

**Table 4:** Biochemical parameters of horseradish (*Armoracia rusticana*) roots and leaves.

Roots				
Parameter	Control	Joint Cultivation	Se	Se + Joint Cultivation
Dry matter, %	23.4 ± 2.1 ab	26.8 ± 2.2 a	26.7 ± 2.3 a	20.6 ± 2.0 b
Nitrates, mg kg^−1^ d.w.	369 ± 32 b	457 ± 41 a	386 ± 33 ab	324 ± 30 b
Ascorbic acid, mg 100 g^−1^ d.w.	159 ± 14 b	224 ± 21 a	237 ± 22 a	247 ± 21 a
AOA *, mg GAE g^−1^ d.w.	17.4 ± 1.1 a	17.2 ± 1.0 a	17.6 ± 1.1 a	19.3 ± 1.2 a
TP **, mg GAE g^−1^ d.w.	7.9 ± 0.5 b	9.2 ± 0.6 a	9.3 ± 0.6 a	10.2 ± 0.9 a
Monosaccharides, % d.w.	5.5 ± 0.5 b	5.7 ± 0.5 b	8.7 ± 0.7 a	8.0 ± 0.7 a
Total sugar, % d.w.	23.6 ± 2.1 a	22.3 ± 2.0 a	19.8 ± 1.7 a	22.5 ± 2.0 a
**Leaves**				
Dry matter, %	26.0 ± 2.3 a	15.7 ± 1.3 b	22.5 ± 2.1 a	15.7 ± 1.3 b
Nitrates, mg kg^−1^ d.w.	170 ± 15 b	195 ± 16 ab	179 ± 15 b	226 ± 18 a
Ascorbic acid, mg 100 g^−1^ d.w.	255 ± 25 b	349 ± 30 a	380 ± 35 a	376 ± 36 a
AOA, mg GAE g^−1^ d.w.	42.8 ± 4.0 b	60.2 ± 5.8 a	56.8 ± 5.2 a	63.9 ± 6.0 a
TP, mg GAE g^−1^ d.w.	19.1 ± 1.6 a	17.9 ± 1.4 a	19.4 ± 1.6 a	17.9 ± 1.4 a

* AOA: total antioxidant activity; ** TP: total polyphenol content. Along each line, values with the same letters do not differ statistically according to Duncan test at *p* < 0.05.

**Table 5:** Mean month temperature and rainfall during the experiment.

Month	2021		2022	
	Mean Temperature, °C	Rainfall, mm	Mean Temperature, °C	Rainfall, mm
January	0.4	16	0.2	18
February	−1	48	2.7	19
March	3.7	34	1.7	50
April	12.3	30	13	21
May	18.2	60	14.3	98
June	22.4	72		
July	24.4	88		
August	26	9		
September	16.3	90		
October	9.8	57		
November	5.5	29		
December	1.5	33		
